# E/N-cadherin switch mediates cancer progression via TGF-*β*-induced epithelial-to-mesenchymal transition in extrahepatic cholangiocarcinoma

**DOI:** 10.1038/bjc.2011.452

**Published:** 2011-11-08

**Authors:** K Araki, T Shimura, H Suzuki, S Tsutsumi, W Wada, T Yajima, T Kobayahi, N Kubo, H Kuwano

**Affiliations:** 1 Department of General Surgical Science (Surgery 1), Gunma University Graduate School of Medicine, 3-39-22 Showa-machi, Maebashi, Gunma 371-8511, Japan

**Keywords:** cadherin switch, TGF-*β*, epithelial-to-mesenchymal transition, cholangiocarcinoma, cancer progression

## Abstract

**Background:**

Epithelial-to-mesenchymal transition (EMT) is a fundamental process governing not only morphogenesis in multicellular organisms, but also cancer progression. During EMT, epithelial cadherin (E-cadherin) is downregulated while neural cadherin (N-cadherin) is upregulated, referred to as ‘cadherin switch’. This study aimed to investigate whether cadherin switch promotes cancer progression in cholangiocarcinoma (CC).

**Methods:**

CC cell lines were examined for migration, invasion, and morphological changes with typical EMT-induced model using recombinant TGF-*β*1. The changes in E-cadherin and N-cadherin expression were investigated during EMT. We also examined E-cadherin and N-cadherin expression in resected specimens from extrahepatic CC patients (*n*=38), and the associations with clinicopathological factors and survival rates.

**Results:**

TGF-*β*1 treatment activated cell migration, invasion, and fibroblastic morphological changes, especially in extrahepatic CC HuCCT-1 cells. These changes occurred with E-cadherin downregulation and N-cadherin upregulation, that is, cadherin switch. Patients with low E-cadherin expression had a significantly lower survival rate than patients with high E-cadherin expression (*P*=0.0059). Patients with decreasing E-cadherin and increasing N-cadherin expression had a significantly lower survival rate than patients with increasing E-cadherin and decreasing N-cadherin expression (*P*=0.017).

**Conclusion:**

Cadherin switch promotes cancer progression via TGF-*β*-induced EMT in extrahepatic CC, suggesting a target for elucidating the mechanisms of invasion and metastasis in extrahepatic CC.

Epithelial cell–cell junctions preserve the tissue system and promote cellular polarity (Perez-Moreno *et al*, 2003). The junctional complex is composed of tight junctions, adherens junctions, and desmosomes (Tsukita *et al*, 2001; Wheelock and Johnson, 2003). Adherens junctions have a critical role in regulating the activity of the entire junctional complex, and the major adherens junction molecules are cadherins, which are calcium-dependent cell adhesion molecules (Takeichi, 1991). Epithelial cadherin (E-cadherin) is expressed by most epithelial tissues. It was well known that loss of E-cadherin expression is correlated with an invasive and undifferentiated phenotype in many epithelium-derived cancer cells (Jawhari *et al*, 1997; Richmond *et al*, 1997; Hugh *et al*, 1999; Joo *et al*, 2002). Neural cadherin (N-cadherin), another adhesion molecule, is associated with heightened invasive potential in cancer. Neural cadherin is typically expressed by mesenchymal cells. However, some cancer cells inappropriately express N-cadherin, which promotes cellular motility and invasion (Islam *et al*, 1996; Nieman *et al*, 1999; Hazan *et al*, 2000; Derycke and Bracke, 2004). A previous study demonstrated that overexpression of N-cadherin in some carcinomas is correlated with invasiveness as a result of N-cadherin-mediated interactions between cancer cells and stromal cells (Hazan *et al*, 2000). Thus, the loss of E-cadherin expression and gain of N-cadherin expression in cancer cells, sometimes called ‘the cadherin switch’, have functional significance in cancer progression.

Epithelial-to-mesenchymal transition (EMT) was first recognised as a central differentiation process in early embryogenic morphogenesis (Shook and Keller, 2003). Embryonic cells lose their E-cadherin expression and acquire cellular rearrangements for conversion to motile fibroblastic cells that are integral to embryonic development. The cells can migrate from their original position to establish new structures. Numerous reports have proposed and supported EMT as a potent mechanism that enhances the detachment of cancer cells from a primary tumour and their migration into the tumour stroma, vessels, and metastatic sites (Huber *et al*, 2005; Christiansen and Rajasekaran, 2006; Thiery and Sleeman, 2006). The cadherin switch is also essential for increased motility, but is not always required for the morphological changes that accompany EMT (Maeda *et al*, 2005). Some reports have described that N-cadherin expression is more important for cancer metastasis than E-cadherin and other EMT inducers (Islam *et al*, 1996; Nakajima *et al*, 2004).

Cholangiocarcinoma (CC) is derived from the epithelium of the bile duct, and can be divided to intrahepatic CC (IHCC) and extrahepatic CC (EHCC), depending on the carcinogenic site. Extrahepatic CC is a malignant epithelial neoplasm with bile duct epithelial differentiation. It often invades in the lateral direction as intraductal invasion and in the vertical direction as extraductal invasion (Sakamoto *et al*, 1998). Extrahepatic CC also metastasises to regional lymph nodes and liver sites, which are the main prognostic factors in EHCC patients (Nagorney *et al*, 1993; Fong *et al*, 1996). The established radical treatment for EHCC is only surgical resection, for which evidence has been reported (Nagorney *et al*, 1993; Klempnauer *et al*, 1997; Shirai *et al*, 1997; Chamberlain and Blumgart, 2000). However, local and distant recurrences occur in many patients after resection of an EHCC. To improve the prognosis after EHCC resection or of unresectable EHCC, it will be necessary to elucidate the mechanisms for the invasion and metastasis of EHCC. There are few reports that have investigated the progression with regard to the cadherin switch or EMT in EHCC (Sato *et al* 2010). We designed the present study to investigate whether the cadherin switch mediates cellular invasion and metastasis in EHCC cells and tissues.

To elucidate the role of the E/N-cadherin switch in CC cells, we investigated the cellular responses in CC cells using TGF-*β*1-induced EMT. We also investigated the expression of E-cadherin and N-cadherin in EHCC tissues using immunohistochemistry, and statistically analysed the associations with clinicopathological factors.

## Materials and methods

### Cell culture

The human EHCC cell lines HuCCT-1 and TFK-1, and human IHCC cell line HuH-28 were obtained from RIKEN BRC through the National Bio-Resource Project of the MEXT, Japan. The CC cells were cultured in RPMI1960 (HuCCT-1 and TFK-1) or DMEM (HuH-28) containing 10% heat-inactivated FBS, unless otherwise stated. For growth in protein-free medium to evaluate the activities of specific growth factors, FBS was omitted from the medium. The cultures were maintained in a humidified chamber under 95% air and 5% CO_2_ at 37°C. The cells were collected and passaged for experiments with 0.25% trypsin and 0.025% EDTA, and the viability was monitored by Trypan blue exclusion.

### Antibodies and reagents

The following medium components were used: RPMI1960, DMEM, and FBS (Sigma-Aldrich Inc., St Louis, MO, USA). The following primary antibodies were used: anti-TGF-*β*1 (R&D Systems Inc., Minneapolis, MN, USA); anti-Smad2, anti-phospho-Smad2, and anti-Smad4 (Cell Signaling Technology Inc., Beverly, MA, USA); anti-E-cadherin (clone: HECD-1; Takara, Tokyo, Japan); and anti-N-cadherin (clone: 3B9; Invitrogen Corp., Carlsbad, CA, USA). An anti-*β*-actin antibody (Sigma-Aldrich Inc.) served as a control. Recombinant TGF-*β*1 (rTGF-*β*1) was obtained from R&D Systems Inc.

### Proliferation assay

Cell proliferation was measured by an MTT tetrazolium assay. Briefly, CC cells (2 × 10^3^ cells per well) were cultured in 96-well microtiter plates in a total volume of 100 *μ*l per well. After initial cell seeding, the cells were analysed using a Cell Counting Kit (Dojindo Laboratories, Tokyo, Japan). Briefly, 10 *μ*l of the cell counting solution was added and incubated under a humidified 5% CO_2_ atmosphere at 37°C for 2 h. The formazan produced was dissolved in 1 N HCl (100 *μ*l per well), and the absorbances of the wells at 450 nm were obtained using a microtiter plate reader (Becton Dickinson, Franklin Lakes, NJ, USA). All experiments were performed in triplicate.

### Invasion assay

Cell invasion assays were performed using 24-well BD BioCoat Matrigel Invasion Chambers (Becton Dickinson, San Jose, CA, USA) to evaluate the invasive cells. Cholangiocarcinoma cells (5.0 × 10^4^) were placed in the upper chamber, and the lower chamber was filled with 750 *μ*l of RPMI1960 or DMEM supplemented with 10% FBS as a chemoattractant. After 48 h of incubation at 37°C, the cells were fixed with 70% ethanol and stained with hematoxylin and eosin. The cells that invaded through the pores to the lower surface of the filter were counted under a microscope. A total of 10 random fields were counted in triplicate assays.

### Migration assay

Cell motility was measured using 48-well BioCoat Cell Culture Inserts (BD Biosciences Inc., Bedford, MA, USA). Briefly, 5 *μ*g ml^−1^ fibronectin in serum-free medium was placed in each lower chamber, which was separated from the upper chamber by a membrane with 8-*μ*m pores. A single-cell suspension of 5 × 10^4^ CC cells in serum-free medium containing different concentrations of rTGF-*β*1 was placed in each upper chamber. After 24 h of incubation at 37°C, the cells were fixed with 70% ethanol and stained with hematoxylin and eosin. The cells that migrated through the pores to the lower surface of the filter were counted under a microscope. A total of 10 random fields were counted in triplicate assays.

### Immunofluorescence staining and confocal laser microscopy

Cells were seeded onto four-chamber slides, fixed with 4% paraformaldehyde for 30 min, permeabilised with 0.1% Triton-X for 20 min, and blocked with 3% bovine serum albumin for 30 min. The cells were then incubated with a primary antibody for 1 h at 37°C, followed by incubation with an Alexa Fluor-conjugated secondary antibody (Alexa Fluor 488 goat anti-rabbit or Alexa Fluor 594 rabbit anti-rat; Invitrogen Corp.) at 1 : 50 dilution for 40 min at 37°C. Actin filaments were stained with Alexa Fluor-conjugated phalloidin (Invitrogen) for 1 h at room temperature.

### SDS–PAGE and western blotting

Aliquots of cell lysates after various treatments were separated by SDS–PAGE, with equal amounts of protein (20 *μ*g) loaded in each lane. All the protein samples were dissolved in SDS sample buffer (100 mM Tris-HCl pH 8.8, 0.01% bromophenol blue, 36% glycerol, 4% SDS) containing 1 mM dithiothreitol, boiled for 5 min, and separated in 5–20% Tris-Tricine Ready Gels (Bio-Rad, Tokyo, Japan). The separated proteins were electrotransferred to Hybond-enhanced chemiluminescence nitrocellulose membranes (Amersham Pharmacia Biotech, Buckinghamshire, UK). The membranes were blocked with 5% non-fat dry milk in PBS containing 0.1% Tween-20 (PBS-T) for 30 min at room temperature, and then incubated with a primary antibody diluted 1 : 1000 in PBS-T overnight at 4°C. Next, the membranes were treated with horseradish peroxidase-conjugated anti-mouse or anti-rabbit secondary antibodies depending on the primary antibody used. After two washes with PBS-T for 30 min each, the membranes were treated with an enhanced chemiluminescence reagent (Amersham Pharmacia Biotech) and exposed to Amersham High Performance Chemiluminescence Film (Amersham Pharmacia Biotech) to visualise the antibody-bound bands. The densities of the positive bands were determined using Adobe Photoshop (Apple Inc., Cupertino, CA, USA) and normalised by the corresponding density of the *β*-actin band.

### Patients and samples

The immunohistochemical study was performed on samples from 38 patients with EHCC who underwent potentially curative surgery without preoperative therapy at the Department of General Surgical Science (Surgery 1), Gunma University Hospital, between 1995 and 2006. The tumour stage was classified according to the 7th tumour-node-metastasis classification of the International Union Against Cancer (UICC). All the patients signed informed consent forms according to our institutional guidelines. Information on sex, age, stage of disease, and histological factors was extracted from medical records.

### Immunohistochemical staining

Immunohistochemical staining of sections for E-cadherin and N-cadherin expression was performed by a standard streptavidin–biotin peroxidase complex method, as described previously (Kato *et al*, 2002; Fukai *et al*, 2005). Each 4-*μ*m section was deparaffinised, rehydrated, and incubated with fresh 0.3% hydrogen peroxide in methanol for 30 min at room temperature to block endogenous peroxidase activity. After rehydration through a graded series of ethanol solutions, the sections were autoclaved in 10 mM citrate buffer (pH 6.0) at 95°C for 20 min and then cooled to 30°C. After rinsing in 0.1 M PBS (pH 7.4), non-specific binding sites were blocked by incubation with 10% normal rabbit serum for 30 min. The sections were then incubated with anti-E-cadherin and anti-N-cadherin primary antibodies at a dilution of 1 : 50 in PBS containing 1% bovine serum albumin at 4°C overnight. The sections were washed in PBS, incubated with biotinylated anti-mouse IgG for 30 min at room temperature, and finally incubated in a streptavidin–biotin peroxidase complex solution (Nichirei Co., Tokyo, Japan). The chromogen, 3,3′-diaminobenzidine tetrahydrochloride, was applied as a 0.02% solution containing 0.005% H_2_O_2_ in 50 mM ammonium acetate-citrate acid buffer (pH 6.0). The sections were lightly counterstained with Mayer's hematoxylin and mounted. Negative controls were established by replacing the primary antibody with normal rabbit serum. No detectable staining was evident in the negative controls.

### Statistical analysis

For continuous variables, the data were expressed as means±s.e.m. The significance of differences between values was determined using Student's *t*-test. Statistical analysis of the immunohistochemical staining was performed using the Wilcoxon signed-rank test, *χ*^2^-test, Mann–Whitney U-test, and Kruskal–Wallis test. Survival curves for the patients were calculated using the Kaplan–Meier method, and analysed using the Log-rank test. Prognostic factors were examined by univariate and multivariate analyses using a Cox proportional hazards model. All differences were deemed significant at *P*<0.05. All statistical analyses were performed with the JMP software package, version 5.01 (SAS Institute Inc., Cary, NC).

## Results

### Expression of TGF-*β* signalling molecules in CC cells, and TGF-*β*1-induced cell migration, invasion, and morphological change

**Figure 1:**
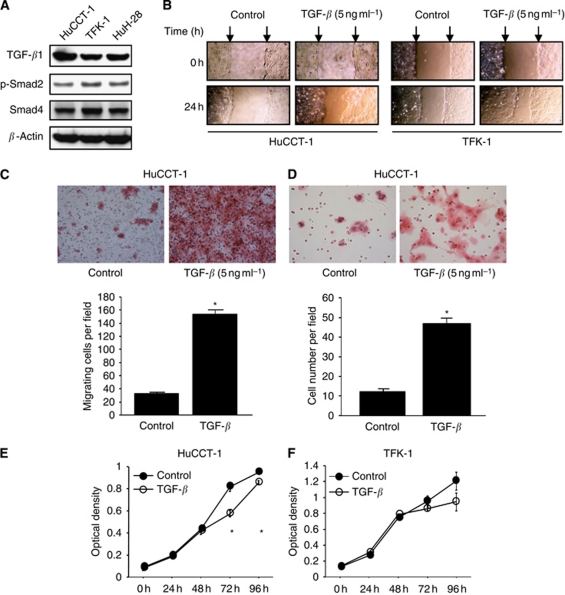
TGF-*β*1 signalling molecule expression and biological activity of rTGF-*β*1 in CC cells. (**A**) Expression of TGF-*β* molecules in CC cells. Total cell extracts were separated by SDS–PAGE using 12% gels, and probed with polyclonal antibodies against TGF-*β*1, phospho-Smad2, and Smad4 to detect their expression. (**B**) Representative examples of wounding experiments in HuCCT-1 and TFK-1 cells cultured with or without rTGF-*β*1 (5 ng ml^−1^). HuCCT-1 and TFK-1 cells were wounded (time 0) and maintained for 24 h in conditioned medium with or without rTGF-*β*1 (5 ng ml^−1^). The arrows point to the edges of the wounds. After 24 h, the wound healing is faster in rTGF-*β*1-treated cells than in untreated cells in both cell lines. (**C**) Migration assays. The mean cell counts (±s.e.m.; *n*=10) of cells that migrated through the pores to the lower surface are shown. HuCCT-1 cells with or without rTGF-*β*1 treatment were tested for migration using a modified Boyden chamber. ^*^P<0.05 *vs* control cells. (**D**) Invasion assays. The mean cell counts (±s.e.m.; *n*=10) of HuCCT-1 cells that invaded through the pores to the lower surface are shown. ^*^P<0.05 *vs* control cells. (**E** and **F**) Proliferation assays. The mean optical densities (±s.e.m.; *n*=3) of the CC cells are shown. HuCCT-1 and TFK-1 cells were cultured on 96-wells with or without rTGF-*β*1 (5 ng ml^−1^). The reagent was injected after 0, 24, 48, 72 or 96 h of culture and the cells were incubated for a further 2 h. The optical densities were detected using a microplate reader. ^*^P<0.05 *vs* 0 h.

Transforming growth factor-*β* not only tends to lose its tumour-suppressive function, but also becomes an oncogenic factor that induces invasion, angiogenesis, EMT, proliferation, and, in certain cases, metastasis (Derynck *et al*, 2001). Transforming growth factor-*β* induces EMT events, such as cellular rearrangement and conversion of cancer cells to motile fibroblastic cells via the TGF-*β* signalling pathway in cooperation with the Ras pathway (Thiery, 2003). Thus, TGF-*β* is well known as a potent inducer of EMT in development and cancer progression. We investigated three CC cell lines in the present study, namely, the HuCCT-1 and TFK-1 cell lines derived from EHCC, and the HuH-28 cell line derived from IHCC. We evaluated the expression of TGF-*β*1 and downstream molecules of the TGF-*β* signalling pathway, Smad2 and Smad4, by western blotting analysis. As shown in Figure 1A, all three CC cell lines expressed TGF-*β*1, phospho-Smad2, and Smad4, suggesting that these cell lines have TGF-*β* production and activation of the TGF-*β* signalling pathway.

Cultured monolayers of fresh HuCCT-1, TFK-1, and HuH-28 cells were wounded with a pipette tip and cultured for a further 24 h in medium with or without rTGF-*β*1. Wound healing was significantly more rapid in the rTGF-*β*1-exposed cells compared with the untreated control cells for HuCCT-1 cells and TFK-1 cells (Figure 1B). We further confirmed the migratory activities using a modified Boyden chamber assay. HuCCT-1 cells were allowed to migrate through the 8-*μ*m pores of polycarbonate filters during *in vitro* culture. As shown in Figure 1C, rTGF-*β*1 induced approximately four-fold increases in the migration of HuCCT-1 cells. We also confirmed the invasive effect using a Matrigel invasion assay, in which rTGF-*β*1 increased the invasive activity of HuCCT-1 cells (Figure 1D).

TGF-*β* has been shown to decrease cell proliferation in various cell types. Therefore, we investigated the effects of exogenous TGF-*β* on the cell proliferation of the CC cell lines *in vitro*. rTGF-*β*1 decreased the cell proliferation in both of the HuCCT-1 and TFK-1 cell lines (Figure 1E and F). These results indicate that TGF-*β*1 is capable of inducing cell motility and decreasing cell proliferation in CC cells.

**Figure 2:**
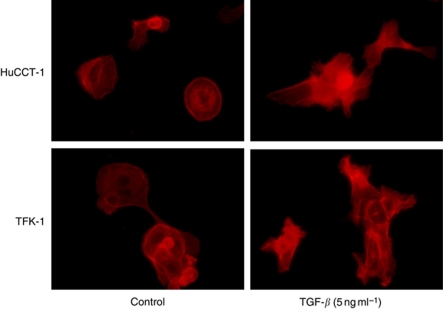
Immunocytochemical analysis of changes in actin filaments in HuCCT-1 and TFK-1 cells in response to rTGF-*β*1 treatment. HuCCT-1 and TFK-1 cells were incubated with rTGF-*β*1 (5 ng ml^−1^) for 24 h. Actin filaments were stained with Alexa Fluor-conjugated phalloidin (1:50; red).

EMT leads to the formation of a migratory mesenchyme that progresses along the primitive streak and populates new areas of the embryo that will develop into the mesoderm and endoderm (Birchmeier *et al*, 1996; Shook and Keller, 2003). Immunocytochemistry confirmed that rTGF-*β*1 changed the morphologies of HuCCT-1 and TFK-1 cells, but not HuH-28 cells (Figure 2).

### TGF-*β*1 induces TGF-*β*/Smad signal activation and changes E-cadherin and N-cadherin expression in CC cells

**Figure 3:**
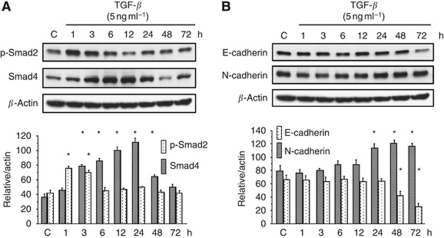
Basal and activation statuses of phospho-Smad2, Smad4, E-cadherin, and N-cadherin in HuCCT-1 cells treated with TGF-*β*. HuCCT-1 cells were stimulated with rTGF-*β*1 (5 ng ml^−1^), and the cells were extracted after 1, 3, 6, 12, 24, 48 or 72 h. The levels of phosphorylated and total proteins were detected by western blotting analysis. C indicates control samples. (**A**) Time-dependent changes in the expression levels of phospho-Smad2, Smad4, and *β*-actin in response to rTGF-*β*1 stimulation. Densitometric analyses of the data are shown under the western blotting bands. ^*^P<0.05 *vs* control cells. (**B**) Time-dependent changes in the expression levels of E-, N-cadherin, and *β*-actin in response to rTGF-*β*1 stimulation. Data represent the means±s.e.m. of triplicate analyses.

It has been shown that TGF-*β* induces EMT through TGF-*β*/Snail activation in CC cells. We examined whether TGF-*β* treatment activated downstream molecules of the TGF-*β* signalling pathway in CC cells. Treatment with rTGF-*β*1 (5 ng ml^−1^) significantly activated the phosphorylation of Smad2 and production of Smad4 in HuCCT-1 cells, as evaluated by western blotting analysis (Figure 3A).

The changes in the expression of E-cadherin and N-cadherin as a result of TGF-*β* treatment were also examined by western blotting analysis. Treatment with rTGF-*β*1 (5 ng ml^−1^) significantly decreased E-cadherin expression and increased N-cadherin expression in HuCCT-1 cells (Figure 3B). However, these changes in E-cadherin and N-cadherin did not occur in HuH-28 cells (data not shown).

### Expression of E-cadherin and N-cadherin in EHCC tissues, and correlations with clinicopathological findings

**Figure 4:**
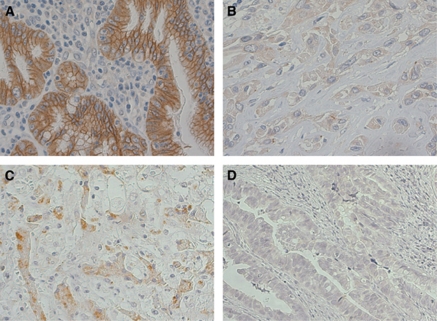
Immunohistochemical staining of E-cadherin and N-cadherin in primary EHCC samples. (**A**) High E-cadherin expression in a primary EHCC. (**B**) Reduced E-cadherin expression in a primary EHCC. (**C**) High N-cadherin expression in a primary EHCC. (**D**) Low N-cadherin expression in a primary EHCC.

Positive staining for E-cadherin in the primary EHCC tissue samples was mainly identified in the cell membrane and the cytoplasm of cancer cells and the benign cholangioepithelium (Figure 4A). Overall, 22 of the 38 EHCC samples (57.9%) had high expression for E-cadherin. Positive staining for N-cadherin in the primary EHCC tissue samples was identified in the cytoplasm of cancer cells and neural bands (Figure 4A). Overall, 9 of the 38 EHCC samples (23.7%) were positive for N-cadherin expression (Figure 4B). The correlations between the clinicopathological characteristics of the EHCC patients and the expression of E-cadherin and N-cadherin are summarised in Table 1. Epithelial cadherin expression was correlated with regional lymph node metastasis (*P*=0.0053), tumour stage (*P*=0.0487), lymphatic invasion (*P*=0.0372), and blood vessel invasion (*P*=0.0131). However, there were no significant associations between N-cadherin expression and the clinicopathological characteristics.

### Univariate and multivariate analyses of prognostic factors, and the prognostic significance in EHCC patients

The results of our univariate and multivariate analyses of prognostic factors in EHCC are shown in Table 2. The parameters that significantly impacted the overall survival were reviewed by the Cox proportional hazards model. In the univariate analyses, venous invasion, lymphatic invasion, TNM stage, and E-cadherin expression status were significantly correlated with survival (Table 2). In the multivariate analysis, E-cadherin expression was not significantly related to survival.

**Figure 5:**
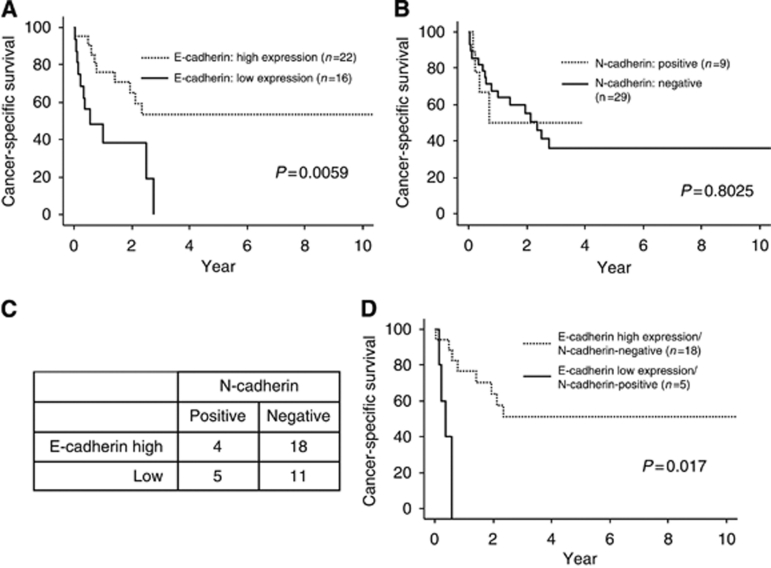
Relationships between postoperative survival and E-cadherin and N-cadherin expression. (**A**) The cancer-specific survival rates at 5 years after surgery were 53.4% for the high E-cadherin expression group and 0% for the low E-cadherin expression group. Kaplan–Meier curves are shown (*P*=0.0059, Log-rank test). (**B**) The cancer-specific survival rates at 5 years after surgery were 0% for the N-cadherin-positive group and 36.3% for the N-cadherin-negative group. Kaplan–Meier curves are shown (*P*=0.8025, Log-rank test). (**C**) Number of patients in the following four subgroups: E-cadherin high expression/N-cadherin-positive; E-cadherin high expression/N-cadherin-negative; E-cadherin low expression/N-cadherin-positive; and E-cadherin low expression/N-cadherin-negative. (**D**) Kaplan–Meier curves of the E-cadherin high expression/N-cadherin-negative and E-cadherin low expression/N-cadherin-positive subgroups are shown (*P*=0.017, Log-rank test). The cancer-specific survival rates at 5 years after surgery were 50.9% for the E-cadherin high expression/N-cadherin-negative group and 0% for the E-cadherin low expression/N-cadherin-positive group.

The cancer-specific survival rate of patients with low E-cadherin expression was significantly lower than that of patients with high E-cadherin expression (*P*=0.0059; Figure 5A). The 5-year cancer-specific survival rate of patients with low E-cadherin expression was 0%, whereas that of patients with high E-cadherin expression was 53.4%.

The cancer-specific survival rates did not differ significantly between the patients with positive and negative expression of N-cadherin (*P*=0.8025) (Figure 5B). The 5-year cancer-specific survival rate of patients with N-cadherin negative expression was 36.3% whereas that of patients with N-cadherin positive expression was 0%.

### Relationships of survival with E-cadherin and N-cadherin statuses in EHCC

As shown in Figure 5C, the EHCC patients were further divided into four subgroups. The 5-year cancer-specific survival rates for the E-cadherin low expression /N-cadherin-positive and E-cadherin high expression/N-cadherin-negative groups were 0% and 50.9%, respectively. Thus, the patients with E-cadherin low expression/N-cadherin-positive expression had a significantly overall lower survival rate than the patients with E-cadherin high expression/N-cadherin-negative expression (*P*=0.017; Figure 5D). These findings emphasise that the E/N-cadherin switch definitely promotes cancer progression and is correlated with a poor prognosis in EHCC patients.

## Discussion

The main findings of the present study are as follows: (1) TGF-*β*1 treatment activated CC cell migration and invasion via EMT, especially in EHCC cells (HuCCT-1); (2) TGF-*β*1 treatment induced fibroblastic morphological changes in EHCC cells (HuCCT-1 and TFK-1 cells); (3) these changes in EHCC cells occurred with decreasing E-cadherin expression and increasing N-cadherin expression, known as the cadherin switch; (4) an immunohistochemical study emphasised that negative E-cadherin expression in EHCC tissues was a prognostic factor for EHCC patients, whereas positive N-cadherin expression was not an independent prognostic factor in EHCC patients; and (5) the change to decreasing E-cadherin expression and increasing N-cadherin expression in EHCC tissues was a strong prognostic factor for EHCC patients.

Changes in cadherin expression may have an important role in the process of EMT and cellular motility (Birchmeier *et al*, 1996). In some malignant tumours, especially undifferentiated tumours and metastases, E-cadherin is mostly negative and N-cadherin is sometimes positive, comprising the cadherin switch (Tomita *et al*, 2000). Nonepithelial cadherins, including N-cadherin, were found to induce a mesenchymal-scattered phenotype associated with reduced E-cadherin and P-cadherin in squamous epithelial cells (Islam *et al*, 1996). In the present study, we first confirmed that the E/N-cadherin switch occurred in CC cells by TGF-*β*-induced EMT in an *in vitro* study. This cadherin switch was related to migratory and invasive effects in CC cells. Furthermore, this function of the E/N-cadherin switch was remarkable, especially in EHCC cells compared with IHCC cells. We also found a prognostic correlation between E-cadherin and N-cadherin expression in EHCC resected tissues in an immunohistochemical study. Neural cadherin may have functional potential to activate progression in EHCC cells. Extrahepatic CC is divided from IHCC based on the anatomical carcinogenic portions. Extrahepatic CC differs from IHCC in its clinical behaviour and progression. For example, EHCC mainly invades along the bile duct epithelium (lateral spreading), whereas IHCC sometimes forms nodules. These characteristics of EHCC and IHCC may be reflected by the differentiation of N-cadherin function observed in this study, showing differentiation between EHCC cells and IHCC cells.

Maeda *et al* (2005) described that downregulation of E-cadherin expression in TGF-*β*1-treated mouse mammary NMuMG cells was much slower than the initiation of morphological changes. This tendency was the same as in our study. The E-cadherin protein levels in HuCCT-1 cells began to decrease at 2 days after rTGF-*β*1 addition. In contrast, the morphological changes of CC cells was prominent within 24 h. Recent reports have pointed out the significance of E-cadherin internalisation as one mechanism that cells use for disruption of E-cadherin function (Palacios *et al*, 2001, 2002; Ivanov *et al*, 2004). However, in the study by Maeda *et al* (2005), internalisation of E-cadherin was not observed, and comparable amounts of E-cadherin remained on the cell surface and were present at cell–cell interfaces in TGF-*β*1-treated NMuMG cells, suggesting that E-cadherin itself does not interfere with the process of EMT and that downregulation of E-cadherin is not essential for morphological EMT to occur. Thus, the cadherin switch regulates in part of migration and invasion, but not morphological changes during EMT in CC cells.

We found that E-cadherin expression was correlated with the prognosis of EHCC patients, similar to the case for many other carcinomas, in an immunohistochemical study. Some E-cadherin expression patterns in cancer tissues have been described, and designated the membranous pattern, cytoplasmic pattern, and negative pattern (Ohno *et al*, 2006). In our immunohistochemical study, E-cadherin was mainly expressed in the cell membrane and the cytoplasm of cells in EHCC tissues. This cytoplasmic pattern of E-cadherin expression was abnormal. This finding suggests that E-cadherin may have functional activity in the cytoplasm of cancer cells, and that E-cadherin expression may be decreased as the cells acquire malignant potential, such as abilities for migration, invasion, and metastasis.

N-cadherin staining was observed in the cytoplasm of CC cells, but did not have a membranous pattern. This pattern may reflect differences in the adhesive nature of the tumour cell population. A previous report described the importance of N-cadherin expression as a key molecule for cancer progression (Derycke and Bracke, 2004). Breast cancer cell lines were found to undergo differentiation from an epithelial phenotype to a mesenchymal phenotype as a result of N-cadherin transfection, without any loss of E-cadherin expression (Hazan *et al*, 1997). In squamous epithelial cells, expression of N-cadherin produced a scattered phenotype with EMT in association with a reduction in E-cadherin (Islam *et al*, 1996). In this study, N-cadherin expression was not an independent prognostic factor for EHCC patients, but the relationship between increasing N-cadherin expression and decreasing E-cadherin expression was a strong prognostic factor for EHCC patients. These findings lead us to propose that N-cadherin has a functional key role in cancer progression, regardless of the protein amounts of N-cadherin, in EHCC.

In conclusion, we have demonstrated that E-cadherin expression and the cadherin switch are correlated with the cancer progression of EHCC cells and the prognosis of EHCC patients through EMT, suggesting a possibility for elucidating the mechanisms of invasiveness and metastasis, which are the main prognostic factors in EHCC patients.

## Figures and Tables

**Table 1:** Clinicopathological characteristics of E-cadherin and N-cadherin in EHCC patients

	**E-cadherin expression**				**N-cadherin expression**		
	**Total**	**High**	**Low**	***P*-value**	**Positive**	**Negative**	***P*-value**
*All patients*	38	22	16		9	29	
							
*Age (years)*				0.2988			0.5598
≤65	20	10	10		6	14	
>65	18	12	6		3	15	
							
*Sex*				0.3764			0.4596
Male	23	12	11		4	19	
Female	15	10	5		5	10	
							
*Histological grading*				0.0769			0.1955
Well diff. (G1)	7	7	6		0	7	
Moderately diff. (G2)	17	9	8		6	11	
Poorly diff. (G3)	10	10	7		3	7	
							
*Tumour size (mm)*				0.3136			>0.9999
36<	22	14	8		5	17	
36⩾	13	6	7		3	10	
							
TNM clinical classification (UICC)							
* pT (primary tumour)*				0.6459			0.1285
T1-2	23	14	5		3	20	
T3-4	15	8	11		6	9	
							
* pN (lymph node metastasis)*				0.0053			>0.9999
N0	26	19	7		6	20	
N1	12	3	9		3	9	
							
* pM (distant metastasis)*				0.3875			>0.9999
M0	37	21	16		9	28	
M1	1	1	0		0	1	
							
*Stage*				0.0487			0.4454
I–II	19	14	5		3	16	
III–IV	19	8	11		6	13	
							
Japanese Society of Biliary Surgery (JSBS) (2003)							
* Hinf*				0.9185			0.7215
(+)	33	19	14		7	26	
(−)	5	3	2		2	3	
							
* Ginf*				0.7353			0.5781
(+)	34	20	14		9	25	
(−)	4	2	2		0	4	
							
* Panc*				0.7285			0.6307
(+)	21	13	8		4	17	
(−)	16	9	7		5	11	
							
* Du*				0.3363			0.7328
(+)	34	21	13		9	25	
(−)	3	1	2		0	3	
							
* Pv*				0.1318			>0.9999
(+)	35	22	13		8	27	
(−)	3	0	3		1	2	
							
*A*				0.333			0.9641
(+)	36	22	14		8	28	
(−)	2	0	2		1	1	
							
* Lymphatic invasion*				0.0372			0.1735
ly 0–1	26	18	8		4	22	
ly 2–3	12	4	8		5	7	
							
* Blood vessel invasion*				0.0131			0.2194
v 0–1	29	20	9		5	24	
v 2–3	9	2	7		4	5	
							
* Peri-neural invasion*				0.2546			0.3017
pn 0–1	6	5	1		0	6	
pn 2–3	30	16	14		9	21	

Abbreviations: A=arterial invasion; diff.=differentiated; Du=duodenal invasion; E-cadherin=epithelial cadherin; EHCC=extrahepatic cholangiocarcinoma; Ginf=gall bladder invasion; Hinf=hepatic invasion; ly=lymphatic invasion; N-cadherin=neural cadherin; Panc=pancreatic invasion; PV=portal vein invasion; v=vessel invasion.

**Table 2:** Univariate and multivariate analyses of prognostic factors using the by Cox proportional hazards model

**Risk factor**	**Reference factor**	***P*-value**	**Hazard ratio**	**95% CI**
*Univariate analysis*				
T factor (primary tumour)	T1–2 *vs* T2–3	0.0361	3.173	1.078–9.343
N factor (lymph node metastasis)	Negative *vs* positive	0.034	3.182	1.091–9.28
Stage (UICC)	Stage I/II *vs* III/IV	0.0152	4.903	1.357–17.713
Historical grade	Tub1-2 *vs* por	0.0466	3.218	1.017–10.176
ly	Negative *vs* positive	0.0347	4.061	1.106–14.918
v	Negative *vs* positive	0.0023	6.717	1.977–22.821
Hinf	Negative *vs* positive	0.0014	12.062	2.628–55.366
Pv	Negative *vs* positive	0.0111	5.946	1.503–23.518
A	Negative *vs* positive	0.0282	22.913	1.397–375.772
E-cadherin	Low *vs* high	0.0234	5.361	1.132–15.042
N-cadherin	Negative *vs* positive	0.9449	0.962	0.319–2.897
				
Multivariate analysis				
Hinf	Negative *vs* positive	0.0019	94.265	5.351–1660.64
Blood vessel invasion	v0–1 *vs* v2–3	0.012	20.782	1.949–221.55
E-cadherin expression	Low *vs* High	0.2179	0.652	0.225–1.890

Abbreviations: A=arterial invasion; CI=confidence interval; E-cadherin=epithelial cadherin; Hinf=hepatic invasion; ly=lymphatic invasion; N-cadherin=neural cadherin; Pv=portal vein invasion; v=vessel invasion.
